# Mesoscale Modelling of Concretes Subjected to Triaxial Loadings: Mechanical Properties and Fracture Behaviour

**DOI:** 10.3390/ma14051099

**Published:** 2021-02-26

**Authors:** Qingqing Chen, Yuhang Zhang, Tingting Zhao, Zhiyong Wang, Zhihua Wang

**Affiliations:** 1 Institute of Applied Mechanics, College of Mechanical and Vehicle Engineering, Taiyuan University of Technology, Taiyuan 030024, China; chenqingqing_5@163.com (Q.C.); zhangyh951000@163.com (Y.Z.); zhaotingting@tyut.cn (T.Z.); wangzhiyong@tyut.edu.cn (Z.W.); 2 Shanxi Key Laboratory of Material Strength & Structural Impact, College of Mechanical and Vehicle Engineering, Taiyuan University of Technology, Taiyuan 030024, China

**Keywords:** concrete, 3D mesoscopic simulation, triaxial loadings, multiaxial stress state, failure criteria, Ottosen parameters

## Abstract

The mechanical properties and fracture behaviour of concretes under different triaxial stress states were investigated based on a 3D mesoscale model. The quasistatic triaxial loadings, namely, compression–compression–compression (C–C–C), compression–tension–tension (C–T–T) and compression–compression–tension (C–C–T), were simulated using an implicit solver. The mesoscopic modelling with good robustness gave reliable and detailed damage evolution processes under different triaxial stress states. The lateral tensile stress significantly influenced the multiaxial mechanical behaviour of the concretes, accelerating the concrete failure. With low lateral pressures or tensile stress, axial cleavage was the main failure mode of the specimens. Furthermore, the concretes presented shear failures under medium lateral pressures. The concretes experienced a transition from brittle fracture to plastic failure under high lateral pressures. The Ottosen parameters were modified by the gradient descent method and then the failure criterion of the concretes in the principal stress space was given. The failure criterion could describe the strength characteristics of concrete materials well by being fitted with experimental data under different triaxial stress states.

## 1. Introduction

In practical engineering structures, concretes are inevitably subject to biaxial and triaxial stress states [1]. In particular, for some massive concrete structures, such as water gates, dams, concrete offshore platforms, foundations of heavy machinery and nuclear reactors, concretes are in a complex stress state. The three principal stresses of concretes are unequal, and most of them are even different combinations of compressive stresses and tensile stresses. In order to accurately analyse and design these structures, it is necessary to study the mechanical responses and damage evolution characteristics of concretes under multiaxial compressive and tensile stress conditions [2]. Many experimental studies of the mechanical behaviour of concretes under multiaxial triaxial loadings have been carried out by researchers [3,4,5,6]. Su et al. studied the multiaxial mechanical behaviour of foamed concrete under triaxial confining pressures [7]. Javanmardi [8] calculated the strength relationship between the lateral confining stress and uniaxial stress by summarising the experimental data of extensive and systematic multiaxial tests and compared these with the proposed relationships by other researchers [9,10,11,12,13,14,15,16]. Glatz et al. developed a novel, high-temperature and high-pressure triaxial system that is suitable for X-ray computed tomography (CT) scanning to capture the role of effective stress on the fluid distribution of rocks under high triaxial pressures [17]. Watanabe et al. designed a new true triaxial cell to study the initiation and propagation of microfractures in granite under confining pressures [18]. Shang analysed the experimental results of normal air-entrained concretes under a tension–compression state and compared the mechanical behaviour of different types of concretes under the same loading conditions [19]. Wang and Song investigated the mechanical characteristics of mass concrete under a triaxial compression–tension–tension loading condition [1]. The specimens were tested in a specially designed, truly triaxial apparatus (designed by the State Key Laboratory of Coastal and Offshore Engineering), which was capable of developing three independent compressive or tensile forces. It can be noted that most of the experiments were done under triaxial confining pressures, but a few triaxial loading experiments were done under complex tensile and compressive stress states. Because the development of multiaxial tensile test system technology is difficult and has a high cost, and several key technical problems exist in the experiments, to date, only a few works have been dedicated to investigating the mechanical properties of concretes under multiaxial tensile stress states. According to the test results obtained by different researchers, it was shown that the mechanical behaviour of concretes under triaxial loads was affected by many factors, such as the loading rate, stiffness of the testing apparatus and the measuring and testing techniques, such as the adopted loading mode and the friction-reducing means along the loading direction. However, despite the difficult test system technology and the lack of uniform specifications, the mesoscopic modelling that demonstrated good robustness gives reliable and detailed damage evolution processes under complex multiaxial loadings. This is helpful towards further understanding the triaxial mechanisms of damage evolution and the mechanical behaviour of concretes.

As an acceptable, cost-effective and time-efficient alternative for experiments, the mesoscale concrete model with accurate constitutive behaviour provides an effective way to predict mechanical behaviour and understand the damage mechanisms of concretes under multiaxial stress conditions. In particular, with the developments in computer capabilities, mesoscale modelling should be a powerful tool for investigating the mechanical behaviour of heterogeneous materials, such as concretes, at the mesoscale level. Comby-Peyrot et al. [20] also suggested that modelling the heterogeneities of concretes was important for studying the damage initiation and progression and investigating the local deformation mechanisms in concrete. Karavelić et al. [21] used the Gaussian distribution to model the heterogeneity of material properties for the aggregate and cement phases and showed that the numerical model could simulate the salient characteristics of concrete-like responses well. With the application of mesoscale modelling in the study of the concrete structure [15,22], the behaviour of concrete material under multiaxial loadings can be well understood. Many researchers have studied the mechanical properties of concretes under uniaxial and biaxial loadings [21,23,24]. Caballero et al. [25] described a method for the 3D mesostructural analysis of concrete specimens under biaxial loading. The results indicated that both the cracking and failure modes and the envelope were in good agreement with the experimental results. However, due to the imperfections for some mesoscopic models and the lack of uniform specifications for the sample size and experimental apparatus, relatively few numerical simulations of triaxial loadings for concretes have been investigated. Currently, the triaxial compressive conditions are mainly studied, especially for high confining pressures. Tran et al. [26] studied the behaviour of concretes under high-confining pressures through a three-dimensional numerical model. The internal physical mechanism of the random cracks and mechanical behaviour of concretes could be well reflected by the mesoscale modelling. Dupray et al. [27] performed numerical analyses of the experiments of concretes under quasistatic triaxial compression at the mesoscopic scale. The numerical model provided a reproduction of the main characteristics of mechanical behaviour for concretes under high confining pressures, both qualitatively and quantitatively: mortar compaction, damage localisation and the influence of aggregates on the limit state. The results suggested that the numerical modelling was helpful for understanding the mesoscale phenomena and the influence of damage zones on macroscale behaviour.

As mentioned above, the study of the mechanical responses and damage evolution characteristics of concretes under multiaxial compressive and tensile stresses is of great significance for concrete structures in terms of rational design, safe operation and material savings. However, due to the heterogeneity of meso-concrete and the complexity of its internal structure, there are rare systematic and comprehensive numerical simulation studies of concretes under triaxial loadings, especially under the combination of unequal stresses of tension and compression. This study investigated the triaxial mechanical behaviour of 3D mesoscale concretes under various loading conditions, namely, compression–compression–compression (C–C–C), compression–tension–tension (C–T–T) and compression–compression–tension (C–C–T). Meanwhile, the damage evolution within the entire concrete specimens was further discussed. Moreover, rich simulation data were systematically obtained to identify the mechanical responses under different triaxial stress states. In this study, the gradient descent method was adopted to iteratively modify the Ottosen parameters to further modify the failure criteria in the stress space of the concretes. The good fitting between the experimental data and the failure surface verified the validity of triaxial numerical simulations in this study. On one hand, it was shown that the mechanical performance of concrete structures under various complex loadings could be predicted and reasonably checked using the modified Ottosen failure criteria. On the other hand, these simulations provided a foundation for establishing the quantitative relations between the macro-mechanical properties of concretes and the mesoscopic parameters of concrete components.

## 2. The 3D Mesoscale Model of the Concrete

### 2.1. Model Generation

The convex polyhedral aggregates inside concrete specimens were generated through Voronoi diagrams. Each Voronoi cell V_i_ consists of each point P in the region. Based on the 3D Voronoi model, a random scaling factor is introduced to generate the random convex aggregates via a shrinking process, and the ITZ (interfacial transition zone) is generated through an extension process. The mesoscale concrete models are efficiently generated.

To avoid malformed cells, the minimum distance between any two nuclei should be controlled based on Equation (1):

(1) $$\delta_{\min}=\left(1-K\right)\delta_{0}$$

where $\delta_{0}$ is the distance between two nuclei and the value of K=0.2 [28] was adopted to achieve complete randomness regarding the shape of the aggregates.

**Figure 1:**
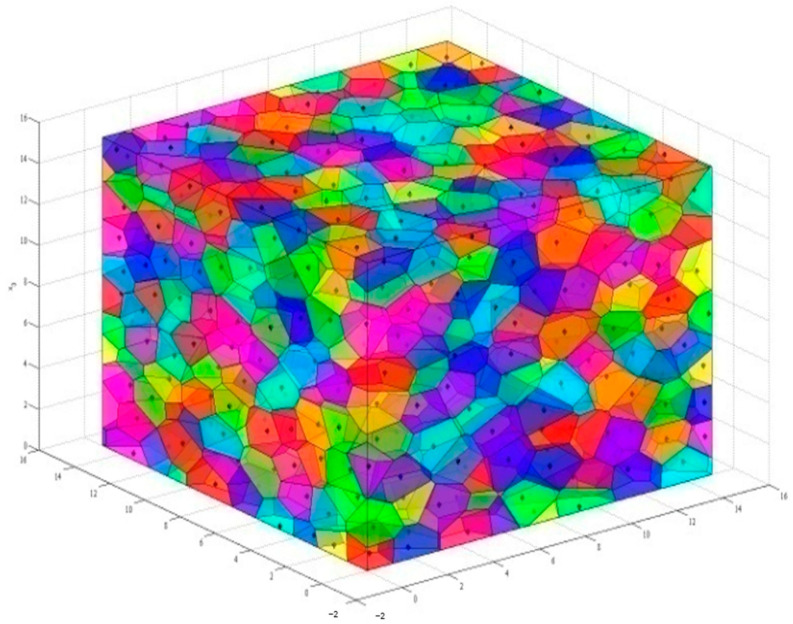
Resulting 3D Voronoi polyhedrons when K = 0.2.

MATLAB (Version R2014a, MathWorks, Natick, MA, USA) [29] was used to establish the initial model of aggregates according to the 3D Voronoi diagram, which is plotted in Figure 1. A random shrinking process was performed to separate adjacent cells. For each cell, the vector $\vec{v}=\left(x,y,z\right)$ from a vertex $p_{i}$ to its corresponding seed $S_{i}$ can be given as $\vec{v}=\left[\begin{array}{c} x_{S_{i}}-x_{p_{i}}\\ y_{S_{i}}-y_{p_{i}}\\ z_{S_{i}}-z_{p_{i}} \end{array}\right]$ and shrunk by the factor q (0<q<1). The updated vertex $p_{i}^{\prime }$ is calculated using Equation (2):

(2) $$p_{i}^{\prime }=p_{i}-q\left|\vec{v}\right|$$

**Figure 2:**
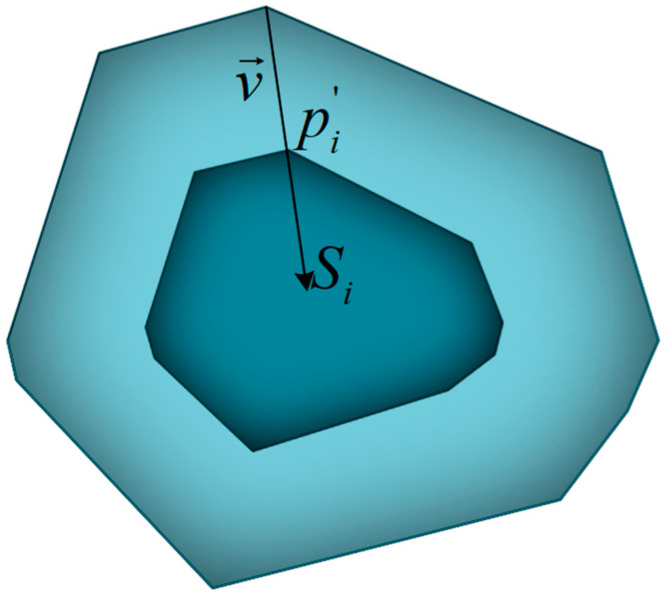
Schematic diagram for a single aggregate.

The shrinking scheme of a single polyhedral cell is shown in Figure 2. In order to achieve the grading requirements, various shrinking factors are employed according to the Fuller curve [30], which is expressed as Equation (3):

(3) $$B\left(d\right)=100{\left(\frac{d}{d_{\max}}\right)}^{n}$$

where B(d) is the total percentage of aggregates that pass the sieve with an aperture diameter d, d_max_ is the maximum diameter of aggregates and n represents an exponent ranging from 0.45 to 0.7, but is generally considered to be 0.5. The aggregate volume within each grading range [d_i_,d_i+1_] can be described as:

(4) $$V_{\mathrm{agg}}\left[d_{i},d_{i+1}\right]=\frac{P\left(d_{i+1}\right)-P\left(d_{i}\right)}{P\left(d_{\max}\right)-P\left(d_{\min}\right)}\times P_{\mathrm{agg}}\times V$$

where $d_{\max}$ and $d_{\min}$ represent the maximum and minimum diameters of the aggregates, $V_{\mathrm{agg}}\left[d_{i},d_{i+1}\right]$ is the aggregates’ volume within the aggregates’ size range $\left[d_{i},d_{i+1}\right]$ and V represents the specimen volume. For the grading range $\left[d_{i},d_{i+1}\right]$, the random shrinking factor q can be given as:

(5) $$q\left[d_{i},\;d_{i+1}\right]=1-\frac{d_{i+1}}{d_{\max}}+\omega \frac{d_{i+1}-d_{i}}{d_{\max}}$$

where ω represents a random variable ranging from 0 to 1.

**Figure 3:**
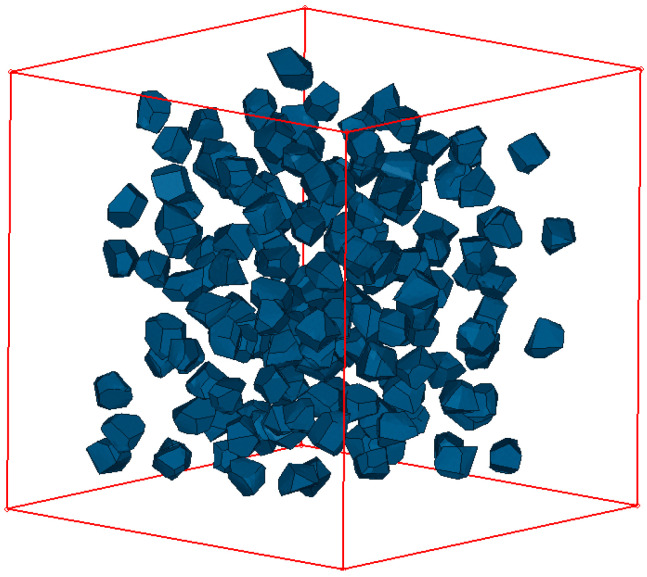
Separated aggregates in a certain space, which were generated using a shrinking process.

**Figure 4:**
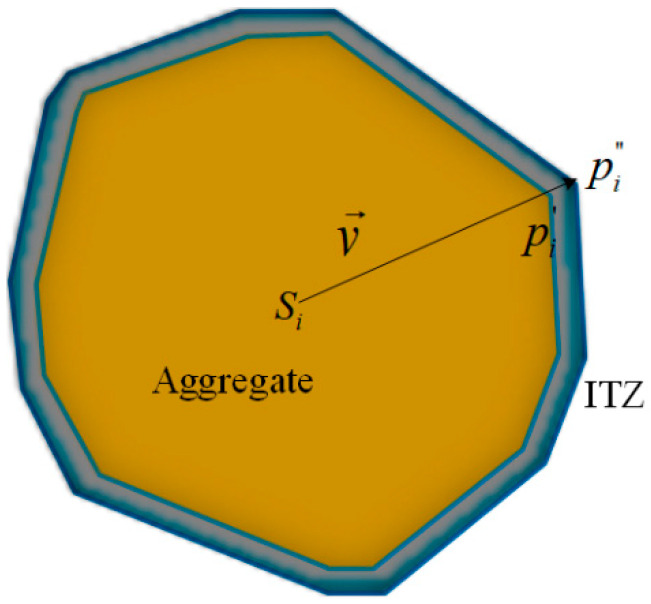
Schematic diagram of generating an interfacial transition zone (ITZ).

Figure 3 shows the modelling results of the random aggregates with the grading sizes. Figure 4 gives the diagram of the ITZ-generating process. Compared with coarse aggregates, the characteristic length of ITZ was very small. Taking into account both the accuracy and computational efficiency, an appropriate thickness of 0.1 mm was employed through an extending process [31,32] and the “free-falling” procedure was adopted to improve the aggregate content [33].

### 2.2. Damaged Plastic Constitutive Model

The heterogeneous constitution, random distribution of phases and arbitrary microcrack patterns all contribute to the complex mechanical behaviour of concretes [34]. The damage plasticity model was used to characterise the irreversible plastic deformations and the stiffness degradation behaviour of the concretes. The model has been well validated for the damage evolution analysis of mesoscopic concretes [35,36,37,38]. Under multiaxial loadings, the effective plastic strain rates of tension and compression are calculated as follows:

(6) $${\dot{\tilde{\varepsilon }}}_{t}^{\mathrm{pl}}=r\left(\hat{\bar{\sigma }}\right){\hat{\dot{\varepsilon }}}_{\max}^{\mathrm{pl}}$$

(7) $${\dot{\tilde{\varepsilon }}}_{c}^{\mathrm{pl}}=-\left(1-r\left(\hat{\bar{\sigma }}\right)\right){\hat{\dot{\varepsilon }}}_{\min}^{\mathrm{pl}}$$

(8) $$r\left(\hat{\bar{\sigma }}\right)=\frac{\sum_{i=1}^{3}{\hat{\bar{\sigma }}}_{i}}{\sum_{i-1}^{3}\left|{\hat{\bar{\sigma }}}_{i}\right|};0\leq r\left(\hat{\bar{\sigma }}\right)\leq 1$$

where ${\hat{\dot{\varepsilon }}}_{\max}^{\mathrm{pl}}$ and ${\hat{\dot{\varepsilon }}}_{\min}^{\mathrm{pl}}$ are the maximum and minimum eigenvalues of the plastic strain rate tensor ${\dot{\varepsilon }}^{\mathrm{pl}}$; $r\left(\hat{\bar{\sigma }}\right)$ represents a stress weight factor.

The yield condition proposed by Lee and Fenves [39,40] was used in the plastic damage concrete model to account for the different strength evolutions under different loadings. For effective stresses, the form of the yield function is:

(9) $$F=F\left(\bar{\sigma },{\tilde{\varepsilon }}^{\mathrm{pl}}\right)=\frac{1}{1-\alpha }\left[\bar{q}-3\alpha \bar{p}+\beta \left({\tilde{\varepsilon }}^{\mathrm{pl}}\right)\langle {\bar{\sigma }}_{\max}\rangle -\gamma \langle -{\bar{\sigma }}_{\max}\rangle -{\bar{\sigma }}_{c}\left({\tilde{\varepsilon }}_{c}^{\mathrm{pl}}\right)\right]=0$$

where α and γ are dimensionless material constants, $\bar{p}$ is the effective hydrostatic pressure, $\bar{q}$ is the Mises equivalent effective stress, and ${\hat{\bar{\sigma }}}_{\max}$ is the algebraical maximum eigenvalue of the effective stress tensor. Furthermore:

(10) $$\beta =\frac{{\bar{\sigma }}_{c}\left({\tilde{\varepsilon }}_{c}^{\mathrm{pl}}\right)}{{\bar{\sigma }}_{t}\left({\tilde{\varepsilon }}_{t}^{\mathrm{pl}}\right)}\left(1-\alpha \right)-\left(1+\alpha \right)$$

(11) $$\alpha =\frac{\sigma_{b0}-\sigma_{c0}}{2\sigma_{b0}-\sigma_{c0}}$$

where ${\bar{\sigma }}_{t}$ and ${\bar{\sigma }}_{c}$ are the effective tensile and compressive cohesion stresses, respectively. The coefficient α can be calculated using the initial equibiaxial yield stress $\sigma_{b0}$ and the uniaxial compressive yield stress $\;\sigma_{c0}$.

When ${\hat{\bar{\sigma }}}_{\max}<0$, the coefficient γ enters the yield function only for the triaxial compressive stress states. γ can be determined by comparing the yield conditions along the tensile meridians (TMs) and compressive meridians (CMs). The corresponding yield conditions are given as Equations (12) and (13). As a typical concrete value, a value of γ=3 gives $K_{c}=\frac{2}{3}$.

(12) $$\left(\frac{2}{3}\gamma +1\right)\bar{q}-\left(\gamma +3\alpha \right)\bar{p}=\left(1-\alpha \right){\bar{\sigma }}_{c},\;(\mathrm{TM})$$

(13) $$\left(\frac{1}{3}\gamma +1\right)\bar{q}-\left(\gamma +3\alpha \right)\bar{p}=\left(1-\alpha \right){\bar{\sigma }}_{c},\;(\mathrm{CM})$$

(14) $$K_{c}=\frac{\bar{q}\left(\mathrm{TM}\right)}{\bar{q}\left(\mathrm{CM}\right)}=\frac{\gamma +3}{2\gamma +3}$$

**Figure 5:**
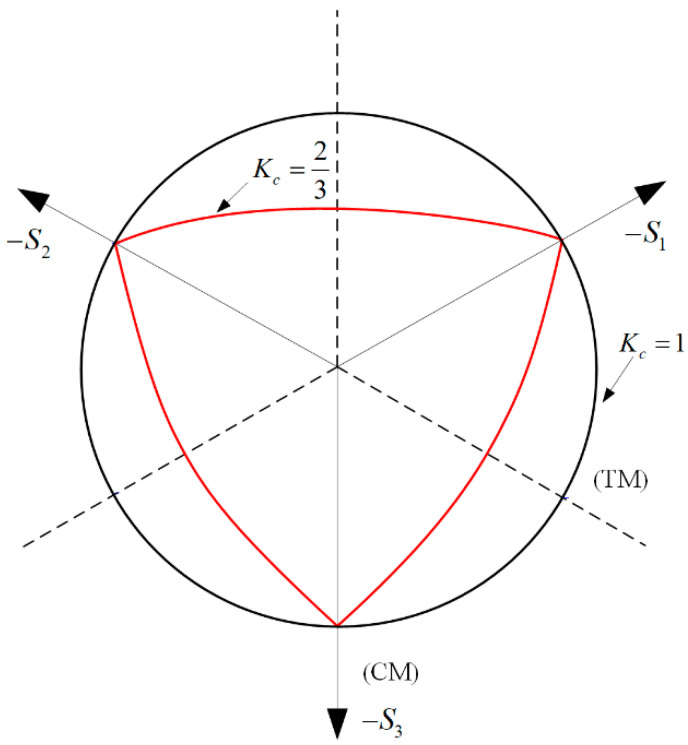
Yield surface in the deviatoric plane, corresponding to different values of K_c_.

**Figure 6:**
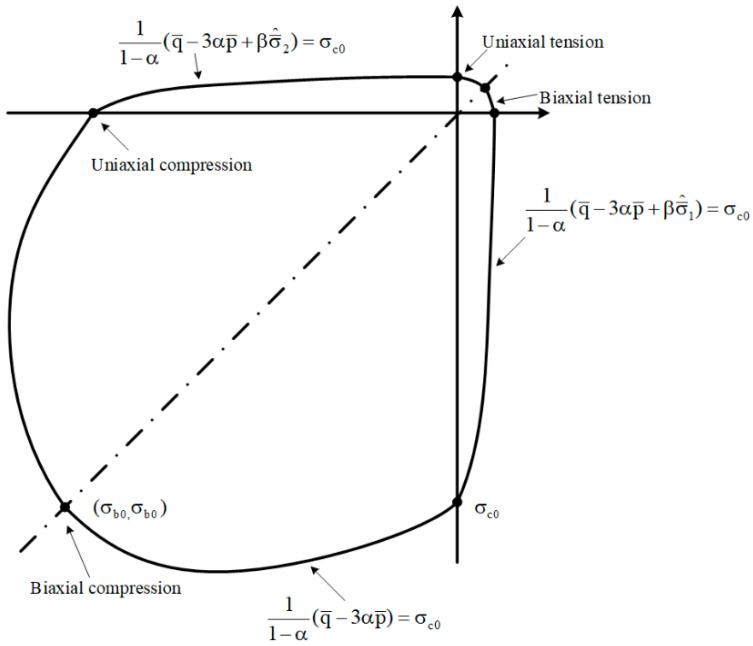
Illustration of the yield surface due to the plane stress, as defined by the concrete damaged plasticity (CDP) model.

Typical yield surfaces are shown in Figure 5 in the deviatoric plane and in Figure 6 for plane stress conditions. For the damaged plastic constitutive model, the flow potential G was chosen to be the Drucker–Prager hyperbolic function:

(15) $${\dot{\varepsilon }}^{\mathrm{pl}}=\dot{\lambda }\frac{\partial G\left(\bar{\sigma }\right)}{\partial \bar{\sigma }},$$

(16) $$G=\sqrt{{\left({\omega \sigma }_{t0}\tan\psi \right)}^{2}+{\bar{q}}^{2}}-\bar{p}\tan\psi ,$$

where ψ is the dilation angle that is measured on the failure curve in the p–q plane at high confining pressures, ω represents the eccentricity and $\sigma_{t0}$ is the uniaxial tensile stress at failure.

## 3. Mechanical Behaviour and Failure Analysis of Concretes under Triaxial Loadings

In this section, the results of the triaxial mechanical behaviour investigation of mesoscopic concretes under various loadings, such as C–C–C, C–T–T and C–C–T, are presented. Then, the damage evolution within the entire concrete specimens is further discussed to clarify the damage mechanism of concretes under triaxial loadings. In this study, the specimen considered was a 25 mm side length cube. The aggregates and mortar phases adopted the mesh size set to 1.0 mm as an average size using tetrahedral elements. For the wedge element used in the ITZ, the element size depended on the contiguous aggregate element, while the size in the thickness direction was an invariable 0.1 mm. An implicit solver was employed to investigate the triaxial loadings using the nonlinear finite element (FE) code ABAQUS/standard (Version 6.14, Dassault Systèmes, Paris, France) [41], where a small time increment had to be used to reduce the influence of the loading kinematics as far as possible. This model is able to precisely control the components, including the geometrical characteristics and the mechanical parameters, and has the advantage of stable convergence by using the implicit algorithm during the simulational processes. The volume fraction of aggregates in the cubic concrete specimens was 37.38%. The aggregate size segment is shown in Table 1. The main computational parameters of the concrete mesoscale contents adopted in the current simulations are given in Table 2. Considering the mortar matrix with a Young’s modulus of 25 GPa, the standard strength of mortar was assumed to be 35 MPa [42]. Due to the limitations of existing measuring techniques, it is hard to clarify the physical and mechanical properties of the ITZ with significant inherent defects in its internal structure. In this study, the ITZ strength was calculated based on the reduction in mortar strength, which can be expressed as the ratio of the ITZ strength to the mortar strength. The ratios adopted by different researchers range from 0.5 to 0.9 [34,43]. One study found that it is reasonable for the equivalent ITZ strength to be 70% of the mortar strength [44]. For the current model of the concretes, the compressive strength of the equivalent ITZ was around 20 MPa, with the Young’s modulus set to 18 GPa. The aggregates’ properties could depend significantly on the natural types. For crushed stones, the Young’s modulus of the aggregates is between 40 and 60 GPa [45]. In the concrete damaged plasticity (CDP) model, the eccentricity was determined using the default value for concretes provided by ABAQUS [41], namely, 0.1. The viscosity coefficient was 1.0 × 10^−5^ [46].

### 3.1. Model Validation under Triaxial Confining Pressures

Under triaxial stress states, the overall strength of concretes of the same specimen size is improved with an increased uniaxial compressive strength f_c_. Therefore, in order to eliminate the influence of different values of f_c_ on the macrostrength, the stress ratios of the axial stress of σ divided by the uniaxial compressive strength of f_c_ can be used to investigate the triaxial mechanical behaviour of concretes.

**Figure 7:**
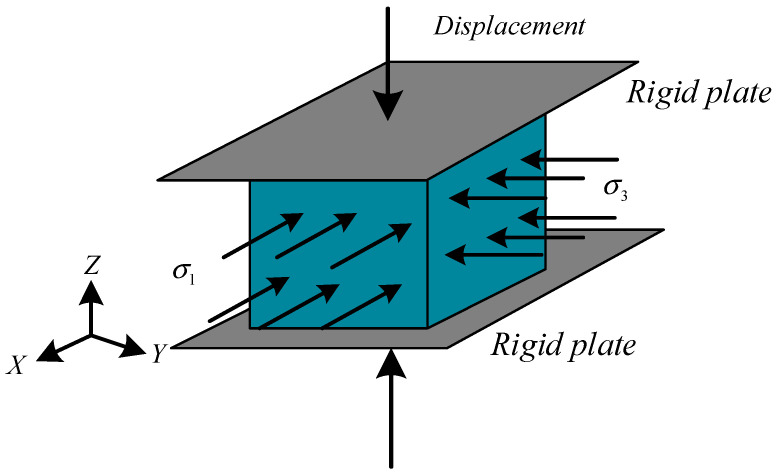
Triaxial compression skeleton diagram.

For numerical simulations of concretes under triaxial loadings, the common loading method is shown in Figure 7, which represents the skeleton diagram of concrete specimens under triaxial loadings. The compressive stresses or tensile stresses are applied in the horizontal direction according to the stress ratio σ_1_/σ_3_ (along the directions of the X-axis (σ_1_) and Y-axis (σ_3_)), and the displacement is applied along the vertical direction (the direction of the Z-axis (σ_2_)). The mesh convergence study was conducted before the loading analysis [32]. An element length of 1 mm was employed in the following analysis.

**Figure 8:**
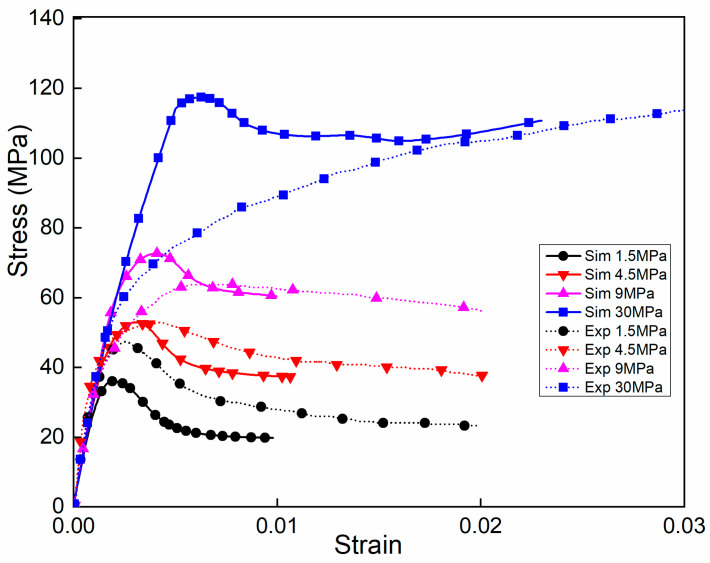
Comparison of the stress–strain curves between the experiments and simulations.

With equal confining stresses (σ_1_/σ_3_ = 1) along the horizontal direction, Figure 8 illustrates the stress–strain curves of experimental tests and numerical simulations under different compressive stresses. Experimental data were obtained from [15], where the confining pressure ranged from 0 to 30 MPa. The confining pressure was applied to the concrete specimens by changing the value of the hydrostatic pressure.

The stress–strain curves changed with the increasing confining pressure. With low confining pressures, the stress–strain curves had clear peak stress points and then experienced the falling stage. Beyond the peak stresses, the softening stages increased with the increasing confining pressures and the stress during the softening stage tended to be stable. Under the confining pressure of 9 MPa, there were no significant descending stages in the curves. When the confining pressure continued to increase to 30 MPa, which was close to the uniaxial compressive strength, the maximum stress of the softening stage could exceed the peak stress, as shown in Figure 8. At that time, the curve went up continuously as the strain increased, but the growth rate of the stress, which is represented by the slope of the curve, decreased gradually and approached 0. Under low confining pressures, the concretes exhibited a brittle fracture feature. With sufficiently large confining pressures, the brittle behaviour of the concretes would disappear, preventing cracks from propagating. In these conditions, the failure of the concretes was driven by the consolidation of the microcracks and the collapse of the micropores, leading to a mechanical response of the brittle materials at the macrolevel. Correspondingly, the numerical simulation results show that the trends of the curves agreed well with the experiments below the confining pressure of 9 MPa. The deviation between the simulation and experimental results fluctuated within a reasonable range at the points of peak stress with increasing confining stress. However, the rising stage of the numerical simulation was slightly different from the experiment under the confining pressure of 30 MPa, which can be explained as follows. Concrete materials are extremely sensitive to confining pressures, which have a great influence on the yield strength of concretes [15]. The increasing confining pressure leads to a change in the principal failure mechanism and substantial flaws or microcracks are inevitably generated inside the initial concretes [47]. The collapse of pores and closure of microcracks during the compacting process result in significantly large strains, up to 3% [5]. In triaxial compressive tests, the mechanical response of concretes presented a clear peak, followed by a relatively smooth decline curve under low confining pressures. Then, with the monotonic increase in confinement, the response was gradually transformed to be ductile, resulting in a plateau. In this study, as a highly nonhomogeneous artificial composite material, the mesoscopic model of the concretes comprised aggregate, mortar and ITZs. The CDP model based on an isotropic damage hypothesis was employed as the constitutive laws of mortar and the ITZ to investigate the inelastic behaviour of concretes. Up to the confining pressure of 30 MPa, the axial strain corresponding to the maximum stress was less than 1%, which was slightly less than that in experiments. The tendencies of the curves and the maximum stresses were close to the experimental results. Consequently, the numerical simulations agreed well with the experiments, which made clear that the mesoscopic model in this study can be used for the mechanical analysis of concretes under triaxial complex stress conditions.

### 3.2. Compression–Compression–Compression Loading

Based on the uniaxial numerical results, the uniaxial compressive strength of the concretes’ f_c_ was 32.3 MPa and the uniaxial tensile strength f_t_ was 2.8 MPa. For the sake of description, the stress ratio of each axial stress to the uniaxial strength, namely, σ_1_/f_c_, σ_2_/f_c_ and σ_3_/f_c_, was adopted to represent the triaxial numerical simulation results. In order to ensure that the specimens were not damaged prematurely, the imposed stresses should not be higher than the uniaxial compressive and tensile strengths. The compressive stress ratios of σ_1_/σ_3_ under various C–C–C loadings are shown in Table 3. There were 25 kinds of compressive loading combinations, where the compressive stresses along the X-axis and Y-axis varied from 0.083f_c_ to f_c_, while the stress ratios along the X and Y directions varied from 0.083 to 12.

**Figure 9:**
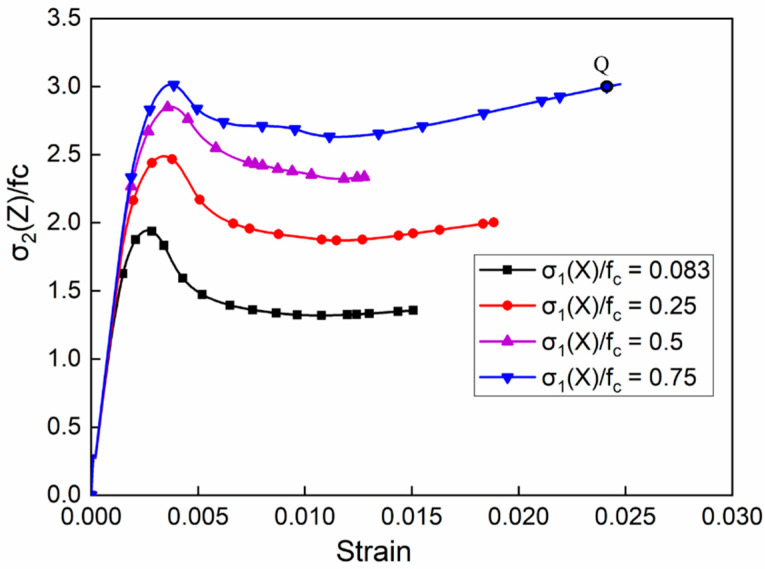
Stress–strain curves of different σ_1_/f_c_ ratios when σ_3_/f_c_ = 0.25.

The stress–strain curves under different compressive stresses of σ_1_ when the compressive stress of σ_3_(Y) was 0.25f_c_ are shown in Figure 9. Compared to the uniaxial compression, the whole strength of the concretes under triaxial confining pressures increased to 2f_c_–3f_c_ and then experienced a small drop after reaching the peak. Keeping the compressive stress along the Y-axis constant, the peak stress increased with the increasing compressive stress along the X-axis. Furthermore, with the increasing stress of σ_1_(X), the softening descending stage was increasingly shorter and the stress platform stage became higher. Because of the strong constraint, the stress was even strengthened with the increasing strain. The tendency of the curves is shown in Figure 9, in which the peak stress was not necessarily the maximum of the curves. After the softening stage, the stress increased to a certain extent and even exceeded the peak stress, as shown at point “Q” in Figure 9. However, the internal structure of the concretes may have begun to be damaged during the process of the stresses increasing after the peak stress for real concretes. For safety reasons, the peak stress was still considered as its compressive strength in the subsequent analysis. After the compressive stress along the X-direction reached 0.5f_c_, the compressive strength was no longer significantly increased.

**Figure 10:**
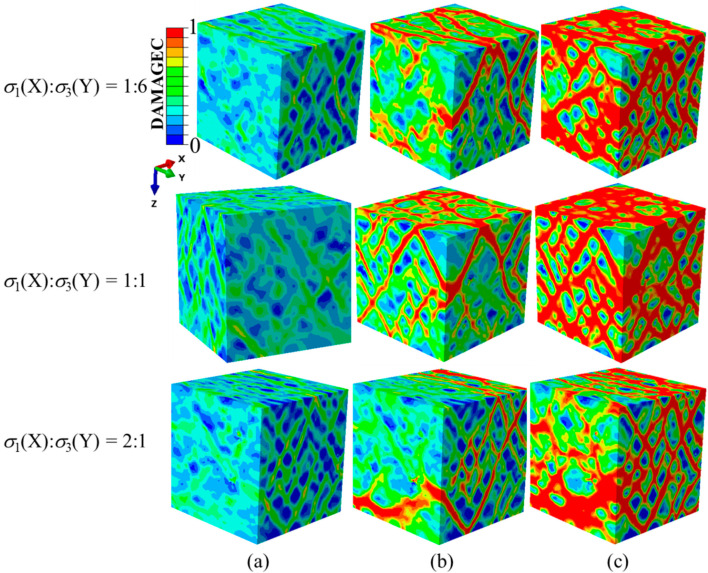
Damage evolution process and failure patterns with different σ_1_/σ_3_ ratios when σ_3_(Y)/f_c_ = −0.25: (**a**) at peak stresses, (**b**) further damage evolution and (**c**) complete failure of the specimens. DAMAGEC denotes damage due to compression.

Figure 10 shows the processes of the damage evolution and failure modes of the concrete specimens under different compressive σ_1_/σ_3_ stress ratios while keeping the stress along the Y-axis constant. It can be seen that no obvious damage zones were formed at the peak stress points. After the peak stress, the damage evolution processes along the Y-axis were basically identical. The damage initiated at the edges of specimen surfaces perpendicular to the Y-axis, then developed obliquely in the Y plane and finally formed intersecting cracks. The damage pattern of the X-Y plane was closely related to the change in the compressive stress ratios. When σ_1_/σ_3_ was less than 1, the generated single cracks were perpendicular to the Z-direction. Single cracks formed into grid cracks as σ_1_/σ_3_ became equal to one and then grid cracks turned into single cracks after σ_1_/σ_3_ was greater than 1. It can be seen that with the increasing stress ratio, the failure mode changed from single cracks to crossing cracks and then to single cracks again.

**Figure 11:**
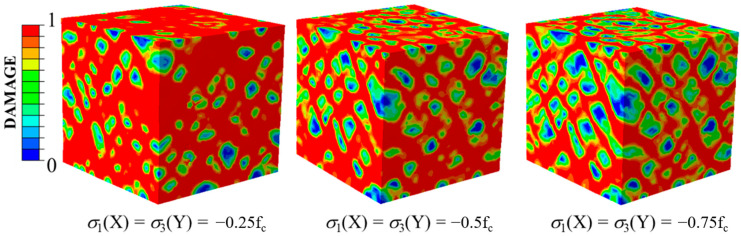
Failure patterns with different compressive stress when ε = 0.01.

The damage modes of the specimens under different compressive stresses with a strain of 0.01 when the stress ratio σ_1_/σ_3_ was 1 are shown in Figure 11. As can be seen, the damage patterns were basically identical and oblique latticed damage zones were formed on the three loading surfaces along the X-, Y- and Z-directions. The main difference between the concretes under different compressive stresses was that the weak lateral restraint was conducive to the damage development with low confining pressure. Basically, the cracks propagated through the whole concrete specimens. With the increasing confining pressure, the lateral restraint was strengthened and the process of damage evolution slowed down.

**Figure 12:**
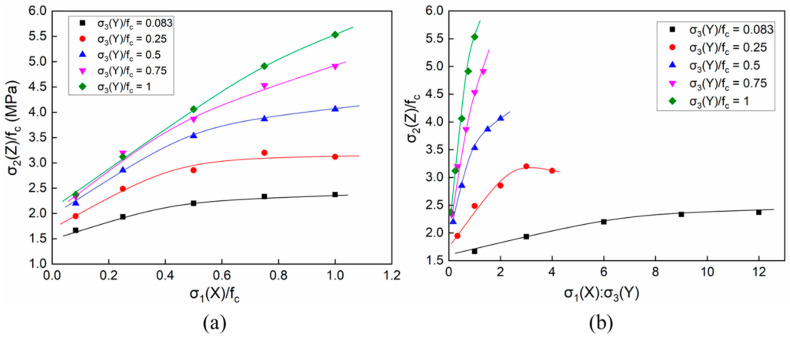
The compressive strength of concrete under triaxial compression: (**a**) σ_1_/f_c_ of the horizontal axis and (**b**) σ_1_/σ_3_ of the horizontal axis.

The results of the 25 groups of numerical simulations are shown in Figure 12, representing the strength of the concretes under triaxial compression. The horizontal axis in Figure 12a is the absolute value of the stress ratio σ_1_/f_c_ and the horizontal axis in Figure 12b is the absolute value of the stress ratio σ_1_/σ_3_. It can be noted that the compressive stress of σ_1_ along the X-axis had an insignificant influence on the growth of concrete compressive strength with low compressive σ_3_(Y) stresses. However, with the increasing compressive σ_3_(Y) stress, the overall strength of the concrete specimens was improved significantly. From the curves, it can be seen that the effects of the lateral compressive stresses on the overall strength could be categorised into one of three situations. The strength of the concretes tended to be relatively constant as the lateral compressive stress was less than 10% of the uniaxial strength. On the other hand, when the lateral compressive stress increased to 50% of the uniaxial strength, the strength of the concretes reached the stress maximum. The strength of the concretes tended to increase linearly when the lateral compressive stress increased to the uniaxial strength, as shown in Figure 12b.

### 3.3. Compression–Tension–Tension Loading

Compared to the triaxial compressive loading, the loading modes of C–T–T involved changing the stresses along the X- and Y-axes, with constant stress along the Z-axis. Table 4 shows various stress of σ_1_(X)/σ_3_(Y) ratios under C–T–T loadings. The tensile stress ranged from 0.02f_c_ to 0.1f_c_ and the stress ratio σ_1_/σ_3_ varied from 0.2 to 5.

**Figure 13:**
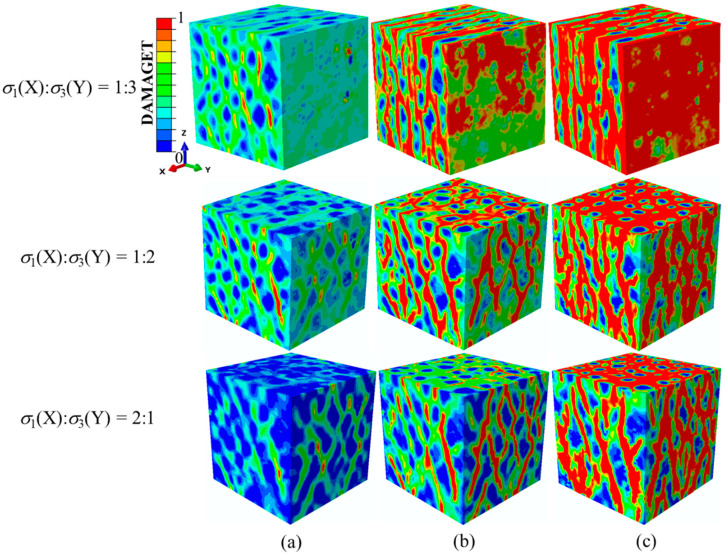
Damage evolution process and failure patterns with different σ_1_/σ_3_ ratios when σ_1_(X)/f_c_ = 0.02 (**a**) at the peak stresses, (**b**) further damage evolution and (**c**) complete failure of the specimens. DAMAGET denotes damage due to tension.

Keeping the tensile stress along the X-direction constant (0.02f_c_), the damage evolution process of the concrete specimens under different σ_1_/σ_3_ stress ratios is shown in Figure 13. It can be observed that there was no significant difference between the damage situations of concrete specimens at the peak stresses, as shown in Figure 13a. During the following process of damage evolution, there were obvious damage bands along the direction of compressive stresses in the Y-Z plane. Furthermore, the damage in the X-Z plane was relatively delayed when the tensile stress along the X-direction was less than that along the Y-direction, as shown in Figure 13b. Then, the damage patterns began to change with the increase of stress ratio σ_3_/σ_1._ When the stress ratio σ_1_(X)/σ_3_(Y) was larger than 1 (σ_1_/σ_3_ > 1), the damage distribution was uniform and the intersecting cracks developed along the X-axis. The final failure modes under different σ_1_/σ_3_ stress ratios can be seen in Figure 13c. With a relatively small stress ratio of σ_3_/σ_1_, the interaction of tensile stresses resulted in damage belts in the X-Z plane developing diagonally. Furthermore, in this circumstance, the X-Z plane was the main failure surface, where the specimen split along the direction of the compressive stress. With the increase of the stress ratio σ_3_/σ_1_, the tensile stresses along the X- and Y-directions interacted with each other and the specimens showed tensile failure with intersecting cracks in each plane.

**Figure 14:**
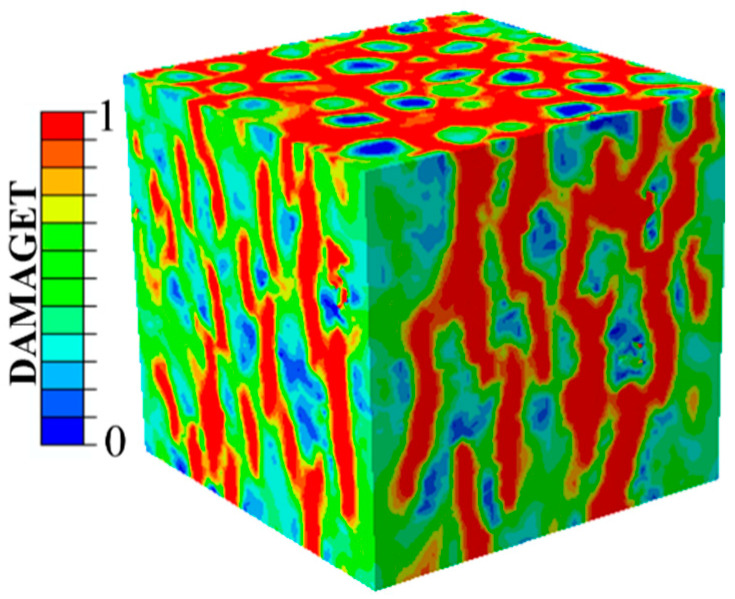
Damage pattern when σ_1_(X)/σ_3_(Y) = 1.

**Figure 15:**
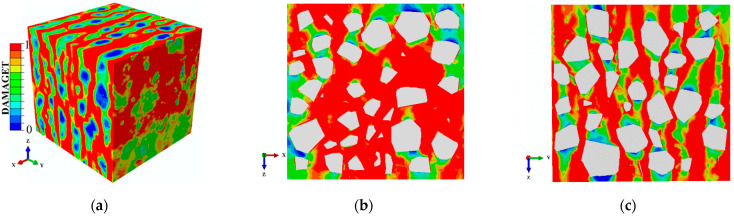
Damage feature when (σ_1_(X)/f_c_)/(σ_3_(Y)/f_c_) = 5: (**a**) a view of the whole specimen, (**b**) a view of the X-Z plane and (**c**) a view of the Y-Z plane.

Under an equal stress condition (σ_1_(X)/σ_3_(Y) = 1), the internal damage pattern of the concrete specimens showed cracks that developed along the boundary of the aggregates in the direction of the compressive stress (the Z-axis), as shown in Figure 14. Figure 15 shows the damage model inside the concretes along the X- and Y-directions when the stress ratio σ_3_(Y)/σ_1_(X) was equal to 5. Obviously, when the applied stresses were not equal (σ_1_(X) ≠ σ_3_(Y)), the specimens showed spallation failure and the cracks were perpendicular to the Y-axis (Figure 15a). The damage belts in the X-Z plane turned into damage zones propagating throughout the whole plane, but the cracks in the Y-Z plane were perpendicular to the Y-direction and propagated along the aggregate boundaries, as shown in Figure 15b,c.

**Figure 16:**
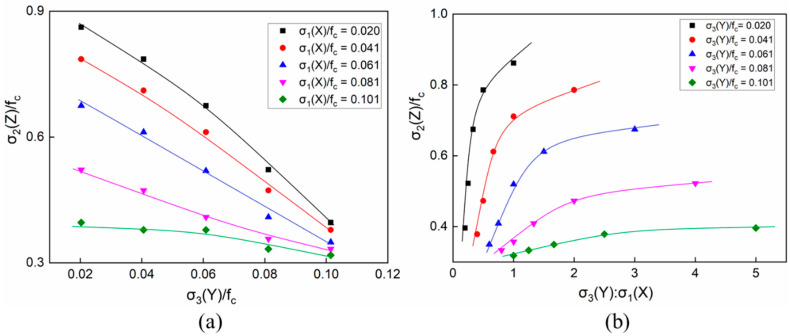
The compressive strength of the concrete under a compressive–tensile–tensile condition: (**a**) σ_3_/f_c_ along the horizontal axis and (**b**) σ_3_/σ_1_ along the horizontal axis.

According to the 25 groups of triaxial loading conditions shown in Table 4, Figure 16 shows the corresponding numerical simulation results. The curves in Figure 16 illustrate the compressive strengths of the concretes under C–T–T loadings, taking the σ_3_/f_c_ and σ_3_/σ_1_ stress ratios as the respective abscissas. With low tensile stresses in the X-direction, the increasing tensile stress in the Y-direction had a significant influence on the overall strength of the concretes, as represented in Figure 16a. It can be seen that the relationship between the overall strength and the compressive stresses along the Y-direction could be approximated as linear. Furthermore, the slopes of the curves under different tensile stresses of σ_1_(X) varied. The whole strength decreased with the increase of σ_3_(Y). In particular, when the concretes were under lower tensile stress for σ_1_(Y), the overall strength witnessed a more significant decline. The trend of the curves in Figure 16b was completely opposite to that in Figure 16a. As the lateral tensile stress ratio σ_3_(Y)/σ_1_(X) increased, the overall strength of the specimens also improved. In particular, when the tensile stress σ_3_(Y) was lower than 0.06f_c_ and the tensile stress ratio was less than 1, the overall strength increased linearly with the stress ratio. After the stress ratio of σ_3_/σ_1_ exceeded 2, the growth of the overall strength slowed down with the increasing stress ratio σ_3_/f_c_. When the tensile stress was relatively high (>0.08f_c_), the overall strength no longer increased with the continuously increasing stress ratio σ_3_(Y)/σ_1_(X) as it approached its extreme strength.

### 3.4. Compression–Compression–Tension Loading

The stress ratios applied to the concretes under triaxial C–C–T loadings are illustrated in Table 5. The X- and Z-axes were the directions of compressive stresses ranging from 0.08f_c_ to f_c_ and the Y-axis was the direction of the tensile stress ranging from 0.021f_c_ to 0.1f_c_. The absolute values of the stress ratios of the compressive stress to the tensile stress were calculated to be 0.8–48.

**Figure 17:**
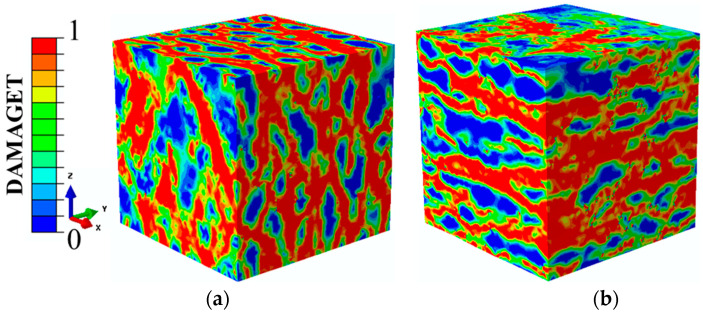
Two failure patterns under compressive-compressive-tensile loadings. (**a**) Y(σ_3_)/f_c_ = 0.21X(σ_1_)/f_c_ = −0.08; (**b**) Y(σ_3_)/f_c_ = 0.21X(σ_1_)/f_c_ = −0.25

Figure 17a,b shows the failure patterns of the concretes when σ_3_ = 0.0210f_c_ and σ_2_ was −0.08f_c_ and −0.25f_c_, respectively. With low compressive stresses, the overall trend of the cracks was perpendicular to the Z-axis. Furthermore, due to the influence of the compressive stresses acting on the specimen, these cracks intersected with each other. In addition, there were also damage zones in the X-Y planes. When the compressive stress continuously increased to 0.1f_c_, the failure modes of the concretes made a difference, as represented in Figure 17b. The failure mode changed from a longitudinal splitting failure to a layered splitting failure. Furthermore, the direction of the main cracks perpendicular to the Y-axis turned towards being perpendicular to the Z-axis.

**Figure 18:**
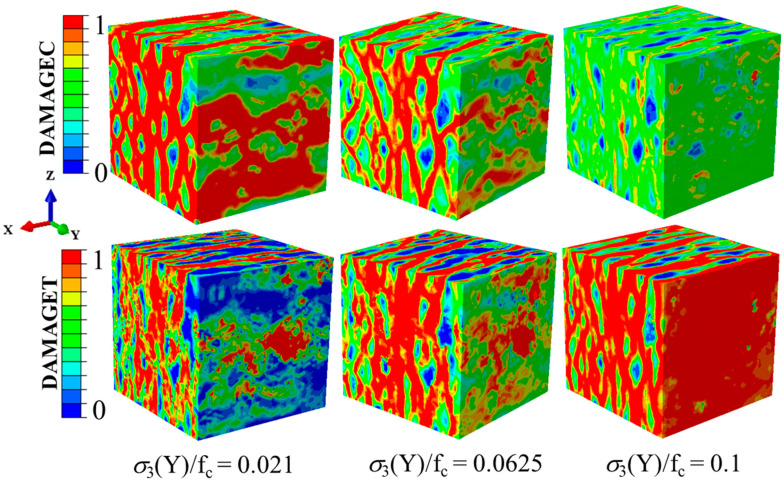
Compressive damage to tensile damage when σ_3_/f_c_ changed under σ_1_(X)/f_c_ = −0.5.

The damage patterns of concrete specimens under different tensile stresses along the Y-direction are shown in Figure 18, where the compressive stress was kept constant along the X-direction at −0.5f_c._ The compressive and tensile damage zones and the failure modes of the concrete specimens were compared. With low tensile stresses, the failure modes were mainly manifested as compression failures, which indicates that the compressive stress was the main cause of the failure. With the increasing tensile stresses, the tensile damage and compressive damage were evenly distributed. This situation meant that at that time, the compressive and tensile stresses worked together to determine the damage development of the specimens. When the tensile stresses increased to the uniaxial tensile strength, there was almost no compressive damage on the surfaces of the specimens and the failure mode was completely caused by the tensile stresses. It can be observed that the stress ratios of the compressive stress to the tensile stress influenced not only the distribution of the damage but also the failure types.

**Figure 19:**
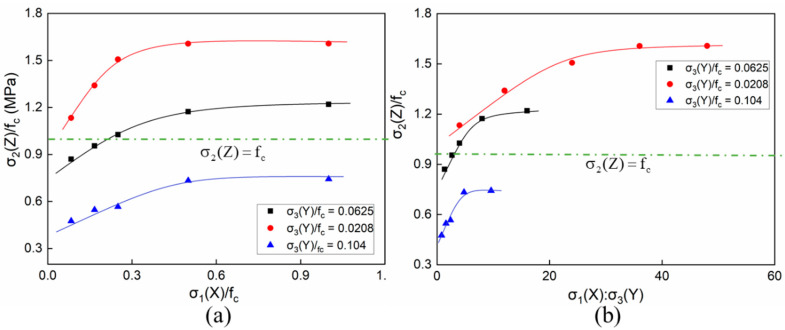
The compressive strength of concrete under compressive–compressive–tensile conditions: (**a**) σ_1_/f_c_ along the horizontal axis and (**b**) σ_1_/σ_3_ along the horizontal axis.

Figure 19 shows the compressive strengths of the concretes under the C–C–T loadings, taking the stress ratios of σ_1_(X)/f_c_ and σ_1_(X)/σ_3_(Y) as the respective abscissas. When the tensile stress was less than 0.0208f_c_, the overall strength under different compressive stresses was above f_c_. Furthermore, the overall strength was below f_c_ as the tensile stress was greater than 0.1f_c_, as shown in Figure 19b. When the tensile stress was close to the median value of the given parameters, which was 0.0625f_c_, the strength varied between 0.8f_c_ and 1.2f_c_ with the increase of the compressive stresses. Obviously, with low compressive stresses less than 0.5f_c_, the strength of the concrete specimens increased with the compressive stresses, as shown in Figure 19a. However, the overall strength tended to be constant after the compressive stress exceeded 0.5f_c_. Then, the whole strength was no longer continuously improved with the increasing lateral compressive stresses.

## 4. Modification of Ottosen Parameters

According to the numerical results discussed above, the strengths of the concretes under multiaxial loadings were a function of the triaxial stress states and could not be predicted only by the independent limits of pure tension, compression and shear stresses. Therefore, in order to correctly estimate the concretes’ strengths, the failure criterion of concrete materials under the combined complex stress states was expressed using a formula. By combining the failure criterion with the constitutive relationship of concrete materials, the mechanical behaviour of concretes can be predicted and then it can be used in engineering analysis and optimisation design under complex multiaxial stress states.

The gradient descent method is widely used in machine learning; it is a common first-order optimisation method which is one of the most simple and classical methods that are used to solve unconstrained optimisation problems. The main purpose of this method is to find the minimum value of the objective function through iteration. The first order means using only the first derivative of the target function and not its higher-order derivatives. Based on the numerical simulation data obtained in Section 3, this study adopted the gradient descent method and took the Ottosen parameter obtained from the classical experimental results as the initial value [48].

The machine learning method was used to update the Ottosen parameters to obtain the yield surface with higher precision. The new partial plane yield curve further validated the numerical simulations of the concretes under triaxial loading that were undertaken in this study. The specific methods and steps are described below.

### 4.1. Calculating the Invariants

Based on the 65 sets of triaxial numerical simulation results obtained in Section 3, the invariants of the stress and deviatoric stress tensors were calculated according to the principal stresses $\left(\sigma_{1},\sigma_{2},\sigma_{3}\right)$ of the above simulations:

(17) $$I_{1}=\sigma_{1}+\sigma_{2}+\sigma_{3}$$

(18) $$I_{2}=\sigma_{1}\sigma_{2}+\sigma_{2}\sigma_{3}+\sigma_{3}\sigma_{1}$$

(19) $$I_{3}=\sigma_{1}\sigma_{2}\sigma_{3}$$

where I_1_, I_2_ and I_3_ are the first, second and third invariants of the stress tensor, respectively. Furthermore,

(20) $$J_{2}=\frac{1}{6}[{\left(\sigma_{1}-\sigma_{2}\right)}^{2}+{\left(\sigma_{1}-\sigma_{3}\right)}^{2}+{\left(\sigma_{2}-\sigma_{3}\right)}^{2}]$$

(21) $$J_{3}=\frac{1}{27}(2I_{1}^{3}-9I_{1}I_{2}+27I_{3})$$

where J_2_ and J_3_ represent the second and third invariants of the deviatoric stress tensor.

In order to meet the geometric requirements of the yield surface of concrete materials, Ottosen [49] proposed a yield function that is denoted by three stress invariants I_1_, J_2_ and θ:

(22) $$f(I_{1},J_{2},\theta )=aJ_{2}+\lambda \sqrt{J_{2}}+bI_{1}-1=0$$

(23) $$\lambda =\{\begin{array}{c} k_{1}\cos[\frac{1}{3}\cos^{-1}(k_{2}\cos3\theta )];\cos3\theta \geq 0,\\ k_{1}\cos[\frac{\pi }{3}-\frac{1}{3}\cos^{-1}(-k_{2}\cos3\theta )];\cos3\theta \leq 0, \end{array}$$

where a, b, k_1_ and k_2_ are constants.

The yield function of the concrete materials was obtained in the Haigh–Westergaard coordinate system, which is defined by the cylindrical coordinates ξ, ρ and θ representing the hydrostatic component, the deviatoric component and the Lode angle component, respectively. These coordinates are functions of the invariants I_1_, J_2_ and J_3_ expressed in terms of the principal stress tensors $\sigma_{1}$, $\sigma_{2}$ and $\sigma_{3}$ as follows:

(24) $$f(\xi ,\rho ,\theta )=\frac{a}{2}\rho^{2}+\frac{\lambda }{\sqrt{2}}\rho +\sqrt{3}b\xi -1=0$$

(25) $$\xi =\frac{1}{\sqrt{3}}I_{1}$$

(26) $$\rho =\sqrt{2J_{2}}$$

(27) $$\cos3\theta =\frac{3\sqrt{3}}{2}\frac{J_{3}}{J_{2}^{3/2}}$$

### 4.2. Fitting Parameters

The gradient of each parameter ($a,b,k_{1}$ and $k_{2}$) is calculated using the chain rule [50]:

(28) $$\frac{\partial f}{\partial b}=I_{1}$$

(29) $$\frac{\partial f}{\partial a}=J_{2}$$

(30) $$\frac{\partial f}{\partial k_{1}}=\cos[\frac{1}{3}\arccos(k_{2}\cos3\theta )]\sqrt{J_{2}}$$

(31) $$\frac{\partial f}{\partial k_{2}}=k_{1}\sqrt{J_{2}}\frac{1}{3}\sin[\frac{1}{3}\arccos(k_{2}\cos3\theta )]\frac{-1}{\sqrt{1-{\left(k_{2}\cos3\theta \right)}^{2}}}\cos3\theta$$

In order to make the fitted parameters more accurate and narrow the range of the iteration values, the gradient descent method needs to give initial values to the unknown parameters. The initial parameter values used for the calculations in this study were derived from [49]. The four initial parameters ($a,b,{\;k}_{1}$ and $k_{2}$) were determined based on the experimental results of four kinds of concrete tests, including the uniaxial compressive strength of f_c_, the uniaxial tensile strength of f_t_, the biaxial compressive strength of f_bc_ and the triaxial stress state at the compression meridian (θ = 60°). Then, the four initial parameters ($a,b,k_{1}$ and $k_{2}$) and the three stress invariants (I_1_, J_2_ and cos3θ) obtained from the results of the numerical simulations in Section 4.1 were substituted into Equations (22) and (23).

In machine learning, the optimal weight parameters can be found based on a certain index, which is used to indicate the current state. The index is defined as the loss function, which represents the unfitting level of the data. In this study, the loss function was defined as follows [51]:

(32) $$\mathrm{loss}=f(I_{1},J_{2},\theta )\neq 0$$

The loss values of all groups were calculated, and then the absolute values of all losses were averaged, which can be expressed as $\bar{\left|\mathrm{loss}\right|}$.

The learning rate of the machine learning lr was set to 0.001 [52]. The updating formula [50] for the parameters is given in Equation (33):

(33) $$P_{\mathrm{new}}=P_{\mathrm{old}}-\mathrm{lr}\cdot \frac{\partial f}{\partial P_{\mathrm{old}}}\cdot \mathrm{loss}$$

The updated parameters were plugged into Equations (22) and (23) again and the yield function $f\left(I_{1},J_{2},\theta \right)$ was iterated 100,000 times to obtain the final values of the updated parameters. The initial and updated parameters, as well as the corresponding loss values, are listed in Table 6. Based on using the original data as the numerical results of the triaxial stress states, it was found that the accuracy of the new parameters obtained using the machine learning method were 4.25 times higher than those of the original parameters (see Table 6).

### 4.3. Validation of the Modified Ottosen Parameters Based on the Triaxial Experimental Tests

The modified Ottosen parameters can provide valuable information, not only for modification purposes of the failure criteria but also for predicting the macroscopic strength of concretes under multiaxial stresses. In order to verify the validity and accuracy of the modified failure criterion, 97 sets of data obtained from the triaxial experimental tests [53] were substituted into the failure criterion. The first step in such a validation was the calculation of invariants based on the experimental data and then the coordinate points (ξ,ρ,θ) were plotted on the yield surface curve on the deviatoric plane and the failure surface in the principal stress space.

The new parameters obtained in Section 4.2 were put into the Haigh–Westergaard stress space coordinate system:

(34) $$f(\xi ,\rho ,\theta )=\frac{a}{2}\rho^{2}+\frac{\lambda }{\sqrt{2}}\rho +\sqrt{3}b\xi -1=0$$

When the yield curve in the partial plane was plotted using the coordinate points (ξ,ρ,θ), the angle θ of the partial plane was divided equally by the angle interval of $\frac{\pi }{120}$. The value of ξ ranged from 0.13 to −10.5 at intervals of 0.3. Then, the calculated coordinate points of (ξ,ρ,θ) were substituted into Equation (34) and the values of ρ in the implicit function were solved.

**Figure 20:**
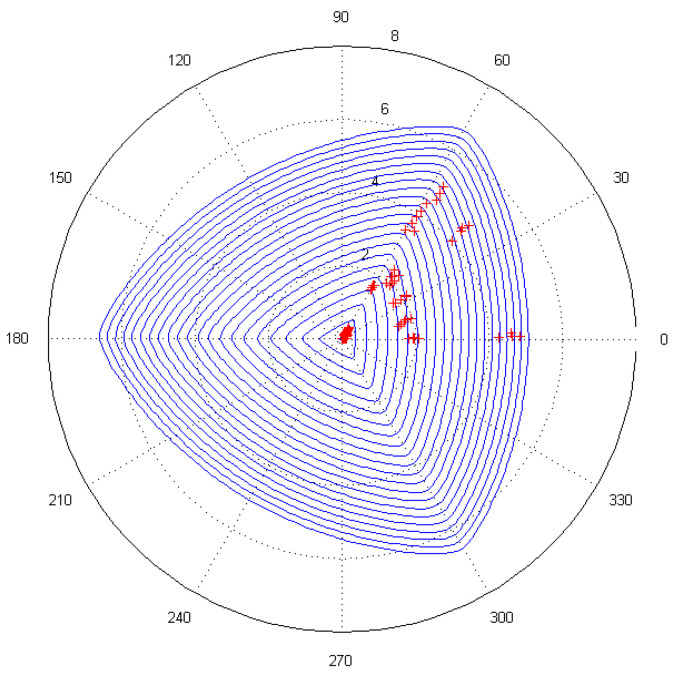
The experimental fitting on the yield surface curve in the deviatoric plane.

The coordinate points of (ξ,ρ,θ), which were obtained using calculations based on the triaxial stresses $\left(\sigma_{1},\sigma_{2},\sigma_{3}\right)$ from the experiments, were plotted on the yield surface curve on the deviatoric plane, which was modified by the Ottosen parameters, as shown in Figure 20. Furthermore, the transformation relationship of the coordinates between the Haigh–Westergaard stress space and the principal stress space is as follows:

(35) $$\{\begin{array}{c} \sigma_{1}=\frac{1}{\sqrt{3}}\xi +\sqrt{\frac{2}{3}}\rho +\cos\theta ,\\ \sigma_{2}=\frac{1}{\sqrt{3}}\xi +\sqrt{\frac{2}{3}}\rho +\cos\left(\theta -\frac{2}{3}\pi \right),\\ \sigma_{3}=\frac{1}{\sqrt{3}}\xi +\sqrt{\frac{2}{3}}\rho +\cos\left(\theta +\frac{2}{3}\pi \right). \end{array}$$

**Figure 21:**
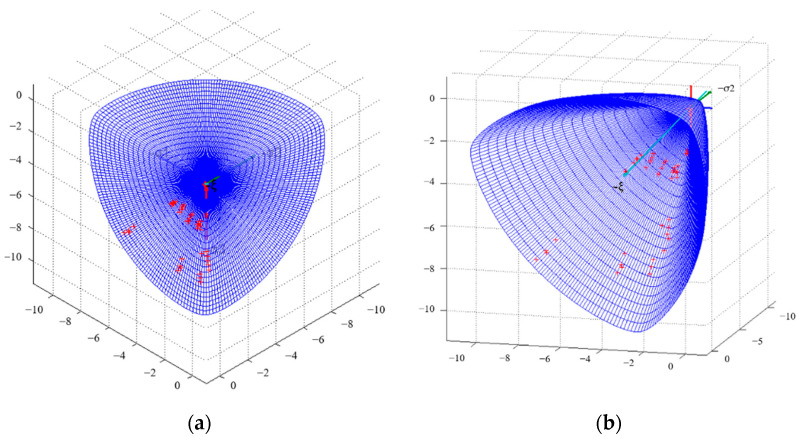
The experimental fitting on the failure surface in the principal stress space: (**a**) overhead view and (**b**) side view.

The coordinates of (ξ,ρ,θ) above were transformed into the coordinates of $\left(\sigma_{1},\sigma_{2},\sigma_{3}\right)$ and these points were plotted on the failure surface in principal stress space, as shown in Figure 21. It can be observed that the experimental data points were basically located on the partial plane curve, which verified the validity of the simulation results of the concrete specimens under triaxial loading. At the same time, the modified parameters were also shown to be reasonable and reliable.

## 5. Conclusions

This study focused on the mechanical behaviour of concretes under triaxial loading conditions and was devoted to characterising the damage evolution of the triaxial behaviour of concrete specimens under various triaxial stress states. Triaxial stresses (including tension and compression) were proportionally applied to the concrete specimens to perform numerical experiments. The following conclusions can be drawn:

(1) The mesoscopic modelling with good robustness gave reliable and detailed damage evolution processes under different triaxial stress states. Rich simulation data were systematically obtained to identify the mechanical responses and damage mechanisms of the concretes. The results show that the multiaxial mechanical behaviour of the concretes was significantly influenced by the level of confining pressures and the lateral tensile stress. Under the combination of tensile and compressive stress, the lateral tensile stresses rapidly aggravated the decline of concrete strength.
(2) The triaxial stress ratios applied to the concrete specimens significantly influenced the damage evolution and failure patterns. Under C–C–C loadings, the direction of the crack propagation mainly depended on the larger lateral compressive stress. Under C–T–T loadings, the initiation and progression of damage belts directly depended on the stress ratios and the intersecting cracks were distributed evenly inside the concretes where the ratio was closer to one. Under C–C–T loadings, the compressive damage inside the concretes gradually transformed into tensile damage with the increasing lateral tensile stress.
(3) The Ottosen parameters were modified using the gradient descent method. The fitting results between the experimental data and the failure surface verified the validity of the triaxial numerical simulations in this study. The validation results indicated that the gradient descent method with good data processing ability was suitable for analysing parameters quickly. Furthermore, the modified Ottosen failure criteria can be used to analyse the mechanical performance of concrete structures under further complex multiaxial stress states.

Finally, further work is needed, where it is likely the influence of mesoscopic components on the macroscopic strength and damage evolution law can be investigated when concretes are under uniaxial and multiaxial loadings, respectively. In the mesoscopic structures of concretes, the identification of the mesoscopic parameters of concrete components is still an open topic. Further investigations on how to establish the quantitative relations between the macromechanical properties of concretes and the mesoscopic parameters should be carried out.

## Figures and Tables

**Table 1:** Size distribution of the aggregates.

Sieve Size (mm)	Total Percentage Retained (%)	Total Percentage Passing (%)
9.5	0	100
4.75	61.53	38.47
2.36	94.84	5.16

**Table 2:** Material parameters of the three-phase materials.

Material	Young’s Modulus (GPa)	Poisson’s Ratio	Compressive Strength (MPa)	Tensile Strength (MPa)
Mortar	25	0.2	35	3.5
ITZ	18	0.2	20	3.0
Aggregate	43	0.23	-	-

**Table 3:** Compressive stress ratios of the X-axis to the Y-axis directions.

$\sigma_{1}\left(X\right):\sigma_{3}\left(Y\right)$		$\sigma_{3}\left(Y\right)/f_{c}$ (Absolute Value)				
		0.085	0.25	0.5	0.75	1
$\sigma_{1}\left(X\right){/f}_{c}$ (Absolute value)	0.083	1	0.3	0.17	0.11	0.083
	0.25	3	1	0.5	0.33	0.25
	0.5	6	3	1	0.67	0.5
	0.75	9	3	1.5	1	0.75
	1	12	4	2	1.3	1

**Table 4:** Tensile stress ratios in the X- and Y-axis directions.

$\sigma_{1}\left(X\right):\sigma_{3}\left(Y\right)$		$\sigma_{3}\left(Y\right)/f_{c}$ (Absolute Value)				
		0.02	0.04	0.06	0.08	0.1
$\sigma_{1}\left(X\right){/f}_{c}$ (Absolute value)	0.02	1	0.5	0.33	0.25	0.2
	0.04	2	1	0.67	0.5	0.4
	0.06	3	1.33	1	0.75	1.67
	0.08	4	2	1.33	1	0.8
	0.1	5	2.5	1.67	1.25	1

**Table 5:** Compressive/tensile stress ratio of the X- and Y-axis directions.

$\sigma_{1}\left(X\right):\sigma_{3}\left(Y\right)$		$\sigma_{3}\left(Y\right)/f_{c}$ (Absolute Value)		
		0.021	0.0625	0.1
$\sigma_{1}\left(X\right){/f}_{c}$ (Absolute value)	−0.08	4	1.33	0.8
	−0.25	12	4	2.4
	−0.5	24	8	4.8
	−0.75	36	12	7.5
	−1	48	16	9.6

**Table 6:** The comparison of the Ottosen parameters and the loss values.

Parameters	a	b	$k_{1}$	$k_{2}$	$\bar{\left|loss\right|}$
Initial value	1.2759	3.1962	11.7365	0.9801	0.2549
Updated value	1.2921	3.2124	11.7527	0.9963	0.0485

## Data Availability

Data is contained within the article.
